# Carcinoembryonic Antigen-Related Cell Adhesion Molecule 5 as a Biomarker for Predicting Response to Erlotinib and Gefitinib in Lung Adenocarcinoma: An Integrative Analysis of Transcriptomic Data of PC-9 and Drug-Resistant PC-9 Cell Lines

**DOI:** 10.3390/ijms27073092

**Published:** 2026-03-28

**Authors:** Pritsana Raungrut, Suchanan Tanyapattrapong, Saowanee Maungchanburi, Kanyaphak Bumrungchoo

**Affiliations:** Division of Biomedical Sciences and Biomedical Engineering, Faculty of Medicine, Prince of Songkla University, Hat Yai 90112, Songkhla, Thailand; bsuchanan.t@gmail.com (S.T.); msaowane@medicine.psu.ac.th (S.M.); kanyaphak.bumrungchoo@gmail.com (K.B.)

**Keywords:** carcinoembryonic antigen-related cell adhesion molecule 5, erlotinib, gefitinib, lung adenocarcinoma, transcriptome

## Abstract

Erlotinib (ER) and gefitinib (GB) are frequently prescribed for patients with advanced lung adenocarcinoma (ADC). Although both ER and GB significantly extend median survival, several patients experience early disease relapse due to treatment resistance. This study aimed to identify common genes associated with acquired resistance to ER or GB. This integrative analysis of transcriptomic data identified differentially expressed genes by comparing sensitive PC-9 cells with ER-resistant (PC-9/ER) or GB-resistant (PC-9/GB) cell lines. A Venn diagram was used to identify common genes. Candidate genes were confirmed in our generated drug-resistant cell lines. After six months, the PC-9/ER and PC-9/GB cell lines exhibited 40.7-fold and 109.2-fold increases in resistance to ER and GB, respectively. Flow cytometric analysis demonstrated that both resistant cell lines were resistant to cell cycle arrest and apoptosis induced by ER or GB. Through integrated analysis, we found that two genes, *KRT13* and *CEACAM5*, were up-regulated in both the PC-9/ER and PC-9/GB cell lines. The expression patterns of these two genes did not align with the transcriptome, with only *CEACAM5* exhibiting consistent gene and protein expressions. Our findings indicate that *CEACAM5* serves as a biomarker for predicting the response of patients to ER and GB treatments.

## 1. Introduction

Lung cancer, a major type of cancer and the leading cause of cancer-related mortality worldwide [1], has two main types: small-cell lung cancer and non-small cell lung cancer (NSCLC, which constitutes 87% of all lung cancer cases) [2]. Despite a reduction in the incidence of NSCLC from 2010–2017, the prevalence of advanced-stage NSCLC is increasing, with an estimated 5-year survival rate of 26.4% [3]. Various options are available for treating advanced-stage NSCLC. One approach is targeted therapy, which employs drugs to treat certain genetic mutations. Mutations in the epidermal growth factor receptor (EGFR) are the most prevalent therapeutically actionable gene mutations in lung cancer [4]. EGFR tyrosine kinase inhibitors (EGFR-TKIs), including first-generation EGFR-TKIs [erlotinib (ER) and gefitinib (GB)] are typically utilized for individuals with EGFR-sensitive mutations as first-line treatment [5]. These drugs obstruct intracellular signaling, thereby preventing cell proliferation, promoting apoptosis, and reducing cell division and growth [6].

Although both ER and GB can significantly prolong the median survival of patients, many experience disease relapse due to treatment resistance [7,8]. In addition, several studies have indicated that over 20% of patients experience early relapse within six months [9,10,11,12]. Therefore, it is crucial to identify potential biomarkers associated with acquired resistance. A few years ago, several studies attempted to identify differentially expressed genes (DEGs) through transcriptome analysis. Some of these studies employed sensitive PC-9 cell lines rather than resistant PC-9 cell lines [13,14,15], because cell lines offer homogeneity and facilitate reproducibility, thereby reducing the confounding effects of cellular heterogeneity and environmental variability often observed with tissue samples [16].

The Gene Expression Omnibus (GEO) currently provides open access to high-throughput genomic data, enabling retrieval of gene expression profiles, including those of PC-9 cells. Nonetheless, these are dependent on diverse variables, such as the platform or fold change in the resistance of the generated resistant cell lines, thereby providing distinct gene markers. Integrative analysis, a set of techniques for combining two or more distinct datasets into a unified dataset for subsequent statistical analysis, is frequently used to identify common genes [17]. Owing to the lack of reports regarding shared genes associated with acquired resistance to both ER and GB, this study identified potential genes associated with acquired resistance to ER or GB through integrated analysis of transcriptome sequencing of sensitive PC-9 cells in comparison with either ER-resistant (PC-9/ER) or GB-resistant (PC-9/GB) cell lines.

## 2. Results

### 2.1. Dataset Characteristics for Identifying DEG

**Figure 1:**
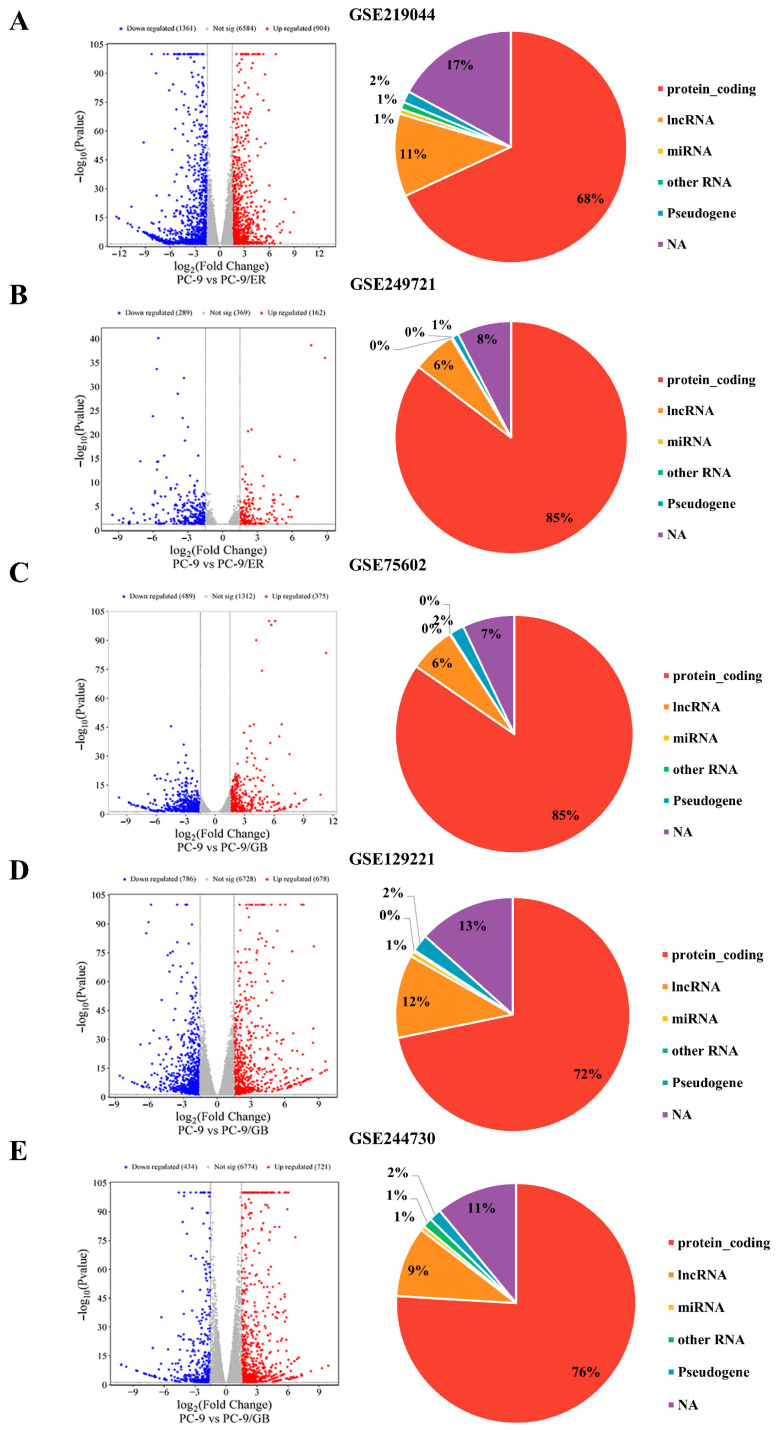
Characteristics of the dataset for PC-9 cells compared to PC-9/ER or PC-9/GB cells. The volcano plot and pie chart depict genes exhibiting significantly different expressions and RNA types for each dataset: GSE219044 (**A**); GSE249721 (**B**); GSE75602 (**C**); GSE129221 (**D**); and GSE244730 (**E**). Red and blue plot dots in the volcano plot represent DEGs with upregulated and downregulated gene expression, respectively.

The inclusion criteria for our dataset selection resulted in the identification of five datasets: two (GSE219044 and GSE249721) for sensitive PC-9 versus PC-9/ER and three (GSE75602, GSE129221, and GSE244730) for sensitive PC-9 versus PC-9/GB. All data were obtained using Illumina sequencing, a prevalent next-generation sequencing technology, including NextSeq 550 or 2000 and HiSeq 2000 or 2500. The resistant cell lines, PC-9/ER and PC-9/GB, exhibited varying fold changes in resistance, ranging from 9.67 to 600, when compared to the sensitive PC-9 cells (Table 1). In the comparison of the sensitivity of PC-9 to PC-9/ER, the DEGs included 1361 upregulated and 904 downregulated genes for GSE219044 (Figure 1A) and 289 upregulated genes and 162 downregulated genes for GSE249721 (Figure 1B). In the comparison of the sensitivity of PC-9 to PC-9/GB, the upregulated DEGs were 489, 786, and 434 genes for GSE75602, GSE129221, and GSE244730, respectively, whereas the downregulated DEGs were 375, 678, and 721 genes for the same datasets (Figure 1C–E). All RNA types across the five datasets were protein-coding, comprising 68–85% of the total RNA types (Figure 1A–E).

### 2.2. Common Gene and Hub Gene Identification

**Figure 2:**
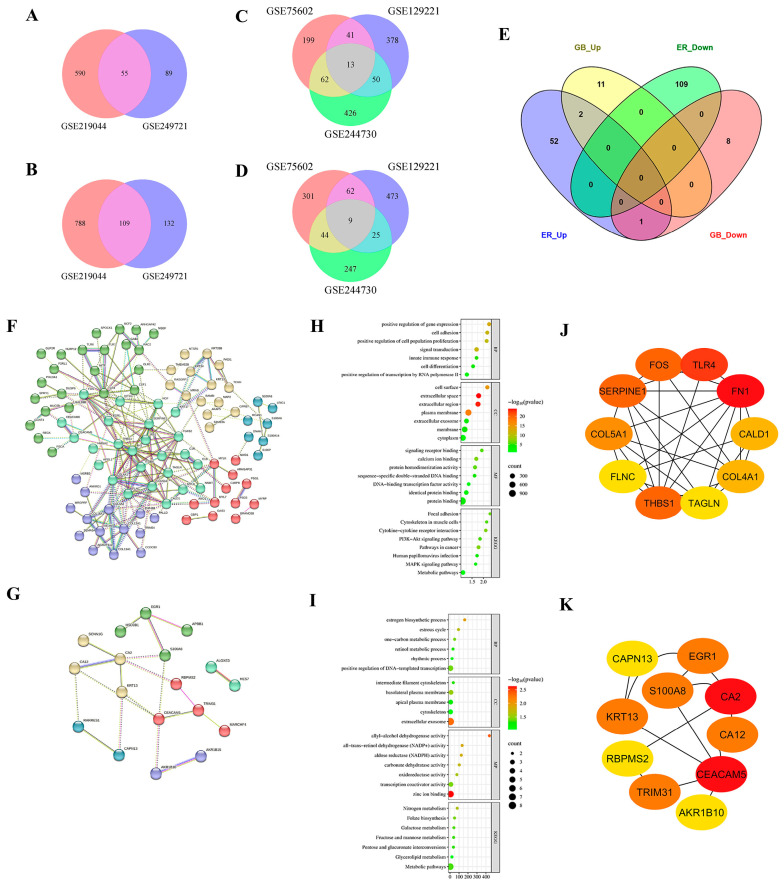
Differentially expressed genes (DEGs) and bioinformatics analysis. Venn diagram illustrating the shared genes between two datasets of up-regulated genes (**A**) and down-regulated genes (**B**) derived from PC-9 versus PC-9/ER cells; Venn diagram depicting three datasets of up-regulated genes (**C**) and down-regulated genes (**D**) from PC-9 compared to PC-9/GB cells; Venn diagram representing the intersection of up-regulated and down-regulated genes of PC-9 in relation to PC-9/ER and PC-9/GB cells (**E**); Cluster analysis of DEGs obtained from PC-9 versus PC-9/ER cells (**F**), PC-9 versus PC-9/GB cells (**G**) using STRING; Gene Ontology (GO) classification and KEGG pathway enrichment analyses of DEGs from PC-9 versus PC-9/ER cells (**H**), PC-9 versus PC-9/GB cells (**I**); Identification of hub genes from PC-9 compared to PC-9/ER cells (**J**) and PC-9 compared to PC-9/GB cells (**K**).

Protein coding genes from each dataset were selected for further investigation using a Venn diagram. We identified the common upregulated and downregulated DEGs for each comparison. In sensitive PC-9 and PC-9/ER cells, 55 genes were upregulated and 109 genes were downregulated, as determined by intersection (Figure 2A,B). In contrast, 13 upregulated genes and 9 downregulated genes were identified as common between sensitive PC-9 and PC-9/GB cells across the three datasets (Figure 2C,D). Furthermore, Venn diagrams illustrate the presence of two upregulated genes, *KRT13* and carcinoembryonic antigen-related cell adhesion molecule 5 (*CEACAM5*), among all datasets, indicating their potential involvement in resistance mechanisms in both ER and GB (Figure 2E). Using bioinformatics tools, all DEGs from each comparison, 164 genes from PC-9 vs. PC-9/ER and 22 genes from PC-9 vs. PC-9/GB, were subjected to further functional genomic analysis. The protein–protein interaction network consisted of 162 nodes and 212 edges for PC-9 compared to PC-9/ER (Figure 2F), and 22 nodes and 20 edges for PC-9 compared to PC-9/GB (Figure 2G). The most enriched Gene Ontology (GO) terms and Kyoto Encyclopedia of Genes and Genomes (KEGG) pathways are shown in Figure 2H and I. Furthermore, we identified 10 hub genes for each comparison, as detailed in Table 2. For ER, two genes were upregulated (*FOS* and *TLR4*), and eight genes were downregulated (*CALD1*, *COL4A1*, *TAGLN*, *COL5A1*, *FLNC*, *FN1*, *SERPINE1*, and *THBS1*) (Figure 2J, Table 3). For GB, nine genes were upregulated (*AKR1B10*, *CA12*, *CA2*, *CAPN13*, *CEACAM5*, *KRT13*, *RBPMS2*, *S100A8*, and *TRIM31*) and one (*EGR1*) was downregulated (Figure 2K, Table 3).

### 2.3. Characterization of Drug-Resistant Cells

**Figure 3:**
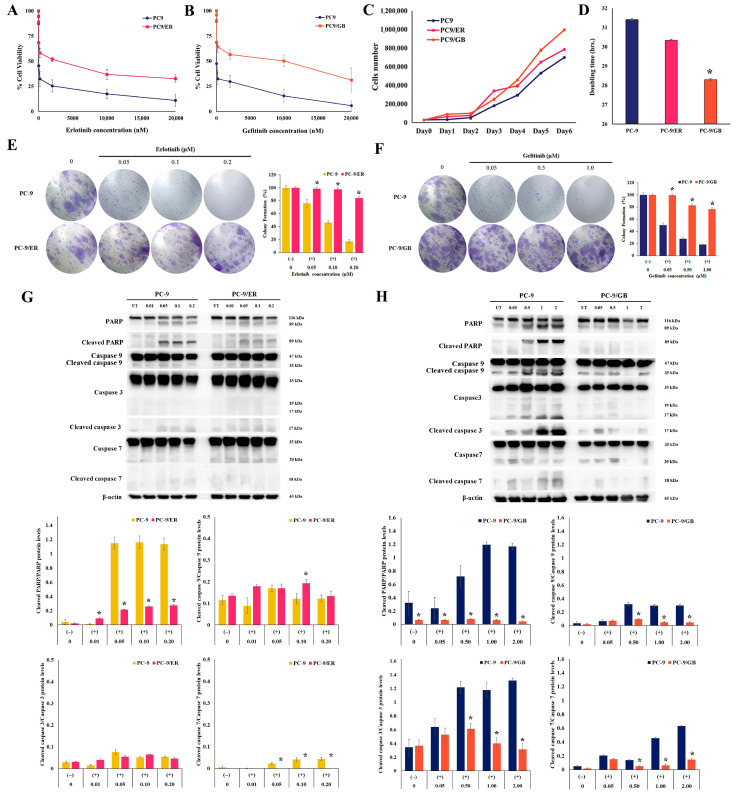
Resistance characteristics of drug-resistant cell lines. Cell viability of PC-9 and PC-9/ER cells following treatment with ER (**A**). Cell viability of PC-9 and PC-9/GB cells following treatment with GB (**B**). Growth curves of PC-9, PC-9/ER, and PC-9/GB cells (**C**). Doubling time of the population for PC-9, PC-9/ER, and PC-9/GB cells (**D**). Colony-forming assay of PC-9 and PC-9/ER cells following treatment with ER at concentrations of 0.05, 0.1, and 0.2 μM (**E**). Colony-forming assay of PC-9 and PC-9/GB cells following treatment with GB at concentrations of 0.05, 0.1, and 1 μM (**F**). Expression levels of apoptosis-related proteins (PARP; cleaved PARP, caspase 9, cleaved caspase 9, caspase 3, cleaved caspase 3, caspase 7, and cleaved caspase 7) and the cleavage ratios of these proteins in PC-9 and PC-9/ER cells following treatment with ER at concentrations of 0.01, 0.05, 0.1, and 0.2 μM (**G**); PC-9 and PC-9/GB cells following treatment with GB at concentrations of 0.05, 0.5, 1, and 2 μM (**H**). * Significant differences were determined using an independent *t*-test (*p* < 0.05).

After more than 6 months of drug resistance development, the resistant cell lines, PC-9/ER and PC-9/GB, were successfully established. Parental PC-9 cells exhibited sensitivity to ER and GB with IC_50_ values of 20.2 nM and 25.4 nM, respectively. In contrast, PC-9/ER and PC-9/GB cell lines subjected to prolonged exposure to ER and GB demonstrated considerable resistance. PC-9/ER cells (IC_50_ = 822.6 nM) displayed a 40.7-fold increase in resistance to ER (Figure 3A), whereas PC-9/GB cells (IC_50_ = 2744.6 nM) demonstrated a 109.2-fold increase in resistance to GB (Figure 3B). The growth rates of PC-9/ER and PC-9/GB cells were higher than those of the parental cells (Figure 3C), whereas the doubling times of PC-9/ER (30.4 h) and PC-9/GB (28.3 h) cells were lower than those of the parental PC-9 cells (31.4 h) (Figure 3D). We assessed the survival of PC-9/ER and PC-9/GB cells using a colony formation assay. After drug treatment, both drug-resistant cell lines exhibited a marked increase in cell survival rates at various concentrations of ER (Figure 3E) and GB (Figure 3F) compared to parental PC-9 cells (*p* < 0.05). In addition, the relevant proteins involved in apoptosis were determined to confirm the resistance of the generated resistant cells. In PC-9/ER, reduced levels of cleaved poly (ADP-ribose) polymerase (PARP) relative to PARP/cleaved caspase 7 relative to caspase 7 were observed at different concentrations of treatment using ER compared to PC-9 cells (Figure 3G). Similarly, PC-9/GB cells exhibited a decrease in cleaved PARP relative to PARP and a reduction in cleaved caspase 9, 3, and 7 relative to their pro-form caspases in comparison to PC-9 cells (Figure 3H).

### 2.4. Effect of ER and GB on Cell Cycle and Apoptosis in Drug-Resistant Cells

**Figure 4:**
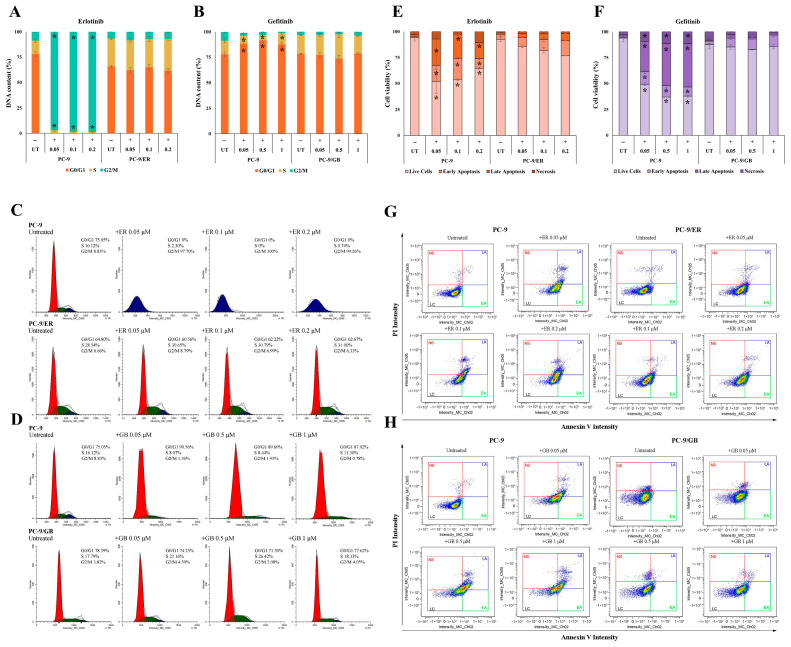
Cell cycle and cell apoptosis analysis by flow cytometry. Cell cycle distribution and flow cytometry histograms of cell cycle phases in PC-9 and PC-9/ER cells following treatment with ER at concentrations of 0.05, 0.1, and 0.2 μM (**A**,**C**); and in PC-9 and PC-9/GB cells following treatment with GB at concentrations of 0.05, 0.5, and 1 μM (**B**,**D**). Apoptosis analysis and flow cytometry plots of apoptotic cell populations in PC-9 and PC-9/ER cells following treatment with ER at concentrations of 0.05, 0.1, and 0.2 μM (**E**,**G**); and in PC-9 and PC-9/GB cells following treatment with GB at concentrations of 0.05, 0.5, and 1 μM (**F**,**H**). * Significant differences were determined using an independent *t*-test (*p* < 0.05).

Flow cytometry was used to further validate the resistance properties of PC-9/ER and PC-9/GB cells by analyzing cell cycle distribution and apoptosis. There was a substantial increase in the G2/M phase population of PC-9 cells treated with ER at various concentrations (8.83% for untreated cells vs. 97.70–100% for treated cells). However, there was no significant change in any of the phases of PC-9/ER cells (Figure 4A,C). Similarly, parental PC-9 cells showed a significant increase in the G0/G1 phase due to GB treatment at different concentrations (75.05% for untreated cells vs. 87.92–90.56% for treated cells). Conversely, PC-9/GB which was subjected to varying concentrations of GB did not exhibit substantial alterations in any of the phases (Figure 4B,D). Apoptosis was evaluated by means of cytometry, similar to the cell cycle assessment. PC-9/ER cells subjected to varying concentrations of ER exhibited an elevated proportion of live cells (76.67–85.49% vs. 51.97–64.68%) and a diminished proportion of apoptotic cells (14.51–23.35% vs. 35.33–48.03%) compared to PC-9 cells (Figure 4E,G). After treatment with GB at different concentrations, PC-9/GB cells showed a markedly higher proportion of live cells (82.57–85.40% vs. 37.01–49.30%) and a substantially lower proportion of apoptotic cells (14.60–17.44% vs. 50.70–62.99%) than their parental cells (Figure 4F,H). These data confirmed that both PC-9/ER and PC-9/GB cells were resistant to ER and GB treatments, respectively.

### 2.5. Expression Levels of Genes and Proteins for Selected Genes

**Figure 5:**
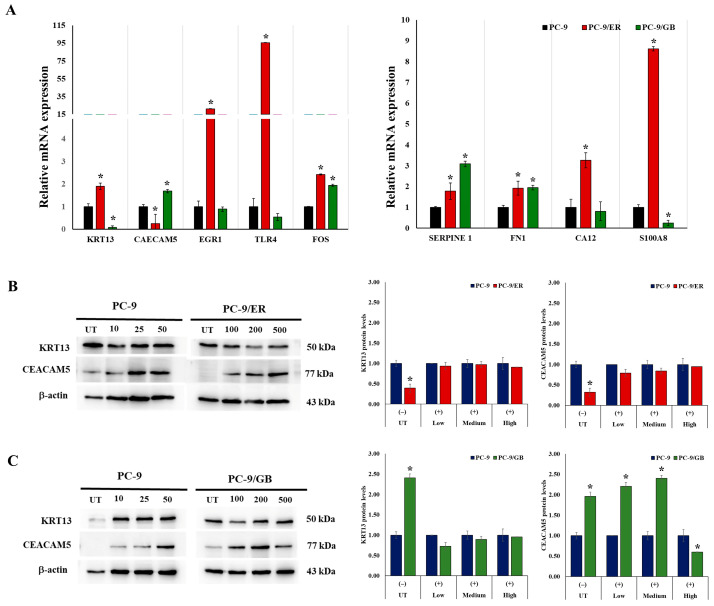
Expression levels of selected genes. Relative mRNA expression levels of *KRT13*, *CEACAM5*, *EGR1*, *TLR4*, *FOS*, *SERPINE1*, *FN1*, *CA12*, and *S100A8* in PC-9, PC-9/ER, and PC-9/GB cells by qRT-PCR (**A**). Expression levels of *KRT13* and *CEACAM5* proteins following treatment with ER and GB at concentrations of 10, 25, and 50 nM for PC-9 and 100, 200, and 500 nM for PC-9/ER and PC-9/GB cells (**B**,**C**). * Significant differences were determined using an independent *t*-test (*p* < 0.05).

By identifying shared genes via a Venn diagram and hub gene analysis, we selected two common genes, *KRT13* and *CEACAM5*, which were upregulated in both PC-9/ER and PC-9/GB. In addition, four upregulated hubs in PC-9/ER (*FOS* and *TLR4*) and PC-9/GB (*CA12* and *S100A8*), as well as three downregulated hubs in PC-9/ER (*SERPINE1* and *FN1*) and PC-9/GB (*EGR1*), were selected for validation using quantitative reverse transcription polymerase chain reaction (qRT-PCR) analysis. These findings indicate that the gene expression levels measured by means of qRT-PCR for the two common genes did not align with the results derived from the transcriptome analysis. *KRT13* and *CEACAM5* cells exhibited substantial increases in PC-9/ER and PC-9/GB, whereas they demonstrated significant decreases in PC-9/ER and PC-9/GB, respectively. Among the other genes identified through hub identification, only two genes (*TLR4* and *FOS*) showed a direction consistent with that of transcriptome analysis, demonstrating higher expressions in PC9/ER cells than in parental PC-9 cells (Figure 5A). Furthermore, we evaluated the protein expression levels of the two common genes using western blotting. These findings indicate that *CEACAM5* displayed a protein expression direction aligned with gene expression, with a decrease in PC-9/ER (Figure 5B) and an increase in PC-9/GB (Figure 5C), whereas *KRT13* did not.

## 3. Discussion

This study utilized integrative analysis to identify shared genes associated with acquired resistance to ER and GB, first-line drugs for patients harboring common EGFR-sensitive mutations. We identified two genes, *KRT13* and *CEACAM5*, that were upregulated in both the PC-9/ER and PC-9/GB cell lines. However, the expression levels of these genes derived from the transcriptome contradicted those obtained using our validation technique.

Difference in the number of identified genes across different transcriptome datasets is common despite being derived from the same cell types. A major contributing factor is the platform, either NextSeq or HiSeq from Illumina which provides varying levels of sensitivity and data resolution. In addition, variation in data processing pipelines, including normalization techniques and sequencing depth, in Illumina sequencing can amplify the impact of fold change selection. Another explanation is the resistant fold change of PC-9/ER and PC-9/GB generated from the parental PC-9 cells within the research group. Some studies used a higher fold change of drug resistance, potentially identifying a more limited subset of genes compared to lower fold change thresholds. Inconsistencies between transcriptome analysis and qRT-PCR validation have been widely recognized. Transcriptomic approaches provide a comprehensive overview of gene expression, whereas qRT-PCR primers focus on specific genes [20]. Variations in normalization strategies, such as the use of internal reference genes in qRT-PCR and global scaling assumptions in RNA-seq also increase the discrepancies between platforms [21]. Therefore, absolute concordance between transcriptome and qRT-PCR data was not expected. In addition, we employed western blotting to validate the protein expression levels of these shared genes. The results indicated that only CEACAM5 had a protein expression level that aligned with the gene expression levels determined by means of qRT-PCR. Gene expression measurements reflect transcript abundance, whereas protein expression is influenced by transcript levels, post-transcriptional regulation, translational efficiency, protein stability, and post-translational modifications [22,23]. Thus, mRNA expression alone is an inadequate predictor of protein expression.

*CEACAM5*, also known as carcinoembryonic antigen (CEA), is a glycoprotein located on the cell surface belonging to the CEA family, a subset of the immunoglobulin superfamily of adhesion molecules. It facilitates cell–cell adhesion via homophilic and heterophilic interactions with other CEACAM family proteins, resulting in cellular communication [24]. *CEACAM5* is abundantly produced in several tissues during fetal development, and its expression is restricted to a few normal cell types [25]. It is significantly overexpressed in various cancers, predominantly colorectal cancer, and is also typically increased in gastrointestinal and pancreatic cancers [26,27]. Elevated *CEACAM5* expression associated with tumor progression and enhanced cell proliferation occurs in breast cancer cells through β-catenin, Cdk4, and mTOR signaling pathways [28]. Clinically, increased *CEACAM5* levels in the serum support its established role as a biomarker for monitoring colorectal cancer [29,30]. Several studies have revealed that *CEACAM5* is a promising target for antibody-based and immune-directed therapies [31].

In lung cancer, *CEACAM5*, which is markedly increased in NSCLC tissues, correlates with tumor stage, lymph node invasion, and histological grade in NSCLC patients. Elevated *CEACAM5* expression enhances cancer cell proliferation and migration, whereas decreased *CEACAM5* expression inhibits NSCLC tumor growth in mouse xenograft models [32]. Additionally, *CEACAM5* functions as a potential receptor for targeted intraoperative molecular imaging resection in lung cancer [33]. Recently, Kim et al. (2023) demonstrated in preclinical studies that therapeutics targeting *CEACAM5*, such as antibody-drug conjugates and *CEACAM5*-targeted CAR-T cells, exhibit cytotoxic effects on *CEACAM5*-expressing NSCLC cells [34]. These studies support our current findings, which demonstrate increased *CEACAM5* expression in PC-9/GB cells at both the gene and protein levels. Furthermore, *CEACAM5* levels were higher in PC-9/GB cells than in PC-9 cells treated with GB at low and medium doses, except at high doses. Conversely, decreased levels of *CEACAM5* were observed in PC-9/ER cells, whereas *CEACAM5* expression remained unchanged following ER treatment in both sensitive and resistant cells. Although ER and GB are first-generation EGFR-TKIs that target identical receptors [6], differential responses were noted within the same cancer cell line, demonstrating context-dependent EGFR inhibition. These differences presumably arise from distinct pharmacokinetic and pharmacodynamic properties, such as intracellular drug accumulation, binding kinetics, and stability, which affect the degree and duration of EGFR signaling suppression [35].

As mentioned above, the findings may indicate a correlation between the expression of *CEACAM5* and the resistance to ER and GB, with varying expression levels serving exclusively as predictive biomarkers in NSCLC patients undergoing treatment with either ER or GB. In GB, elevated *CEACAM5* expression may serve as a biomarker for monitoring drug responses. However, the current investigation was limited by the absence of clinical samples to assess the clinical value of *CEACAM5* as a biomarker for predicting or monitoring drug responses. Further clinical studies are required to validate these findings.

## 4. Materials and Methods

### 4.1. Collection of Transcriptome Data

Gene expression profiles were obtained from the Gene Expression GEO database (http://www.ncbi.nlm.nih.gov/geo/ (accessed on 1 May 2025)) for differential expression analysis. Datasets were selected using the following criteria: (1) inclusion of gene expression profiles for sensitive PC-9 cells and their derived resistant cell lines; (2) generation of PC-9 resistant cells from either PC-9/ER and PC-9/GB cell lines; and (3) reporting the IC_50_ values of both sensitive PC-9 cells and their derived resistant cell lines.

### 4.2. Data Processing of DEGs

Significant DEGs between PC-9 cells and either PC-9/ER or PC-9/GB cells were analyzed using the GEO query and limma R packages [36] using GEO2R in the NCBI-GEO website. Student’s *t*-test was used to identify DEGs in the two sample types. *p*-values were adjusted using the false discovery rate approach according to the Benjamini–Hochberg method. DEGs with a *p* < 0.05, an adjusted *p* < 0.05, and |log2fold change (FC)| > 1.5 were considered significantly different. The RNA types were identified using ShinyGO (https://bioinformatics.sdstate.edu/go/ (accessed on 30 May 2025)).

### 4.3. Bioinformatics Analysis

DEGs, annotated as protein-coding genes, were integrated to identify common genes using Venn diagrams (https://bioinfogp.cnb.csic.es/tools/venny/ (accessed on 15 June 2025)). Cluster analysis was performed using the k-means clustering method in STRING (http://string-db.org/ (accessed on 20 June 2025)) [37]. The web-based Database Annotation Visualization Integrated Discovery (DAVID) platform (https://david.ncifcrf.gov/home.jsp (accessed on 18 July 2025)) was used to obtain biological interpretations related to three main GO categories: biological process, cellular component, and molecular function. KEGG was used to identify relevant pathways. Hub DEGs were identified using the CytoHubba plug-in, with the top 10 nodes ranked according to their degree [37].

### 4.4. Cell Line and Drugs

The PC-9 cell line, which comprised an EGFR exon 19 deletion mutation in a human patient with lung ADC, was acquired from the American Type Culture Collection (ATCC; Manassas, VA, USA). The cells were grown in complete RPMI-1640 medium (Sigma-Aldrich, St. Louis, MO, USA) supplemented with 10% fetal bovine serum (FBS; Gibco, Thermo Fisher Scientific, Waltham, MA, USA) and 1% penicillin-streptomycin (5000 U/mL; Gibco, Thermo Fisher Scientific, Waltham, MA, USA). Cultures were maintained at 37 °C in a humidified incubator with 5% CO_2_. Upon reaching 80–90% confluence, the cells were subjected to trypsinization with 0.25% trypsin-EDTA (Gibco, Thermo Fisher Scientific, Waltham, MA, USA) and subsequently counted using a hemocytometer with 0.4% trypan blue staining (Gibco, Thermo Fisher Scientific, Waltham, MA, USA). ER and GB were purchased from MedChemExpress (Monmouth Junction, NJ, USA), dissolved in dimethyl sulfoxide (DMSO, Gibco, Thermo Fisher Scientific, Waltham, MA, USA), and stored at −20 °C. The aliquots were diluted to the required concentrations before use.

### 4.5. Generation of PC-9/ER and PC-9/GB Cell Lines

A stepwise method was used to generate drug-resistant cell lines as described in our previous study [38]. The IC_50_ values of ER and GB for parental PC-9 cells, which represent the concentrations inhibiting 50% cell growth, were used as the initial doses. A total of 5 × 10^5^ cells were seeded into T25 flasks (Corning Inc., Corning, NY, USA) and incubated for 24 h. Media containing either ER at 20.2 nM or GB 25.4 nM was added, and the cells were incubated for 3 days. Subsequently, the drug-containing medium was replaced with drug-free medium, and the cells were grown until they reached confluence of 70–80%. This cycle was repeated thrice to confirm resistance. Thereafter, the drug concentration was gradually increased over a 6-month period. Resistance characteristics were validated by constructing dose-response curves for PC-9/ER and PC-9/GB cells and comparing them with sensitive PC-9 cells. IC_50_ values were computed and the FC in resistance was determined as the ratio of the IC_50_ of resistant cells to that of sensitive cells.

### 4.6. MTT (3-(4,5-Dimethylthiazol-2-yl)-2,5-diphenyltetrazoliumbromide) Assay

Cells were seeded in 96-well plates at a density of 1 × 10^4^ cells/well and incubated at 37 °C for 24 h. Cells were exposed to ER or GB at concentrations of 1, 2, 20, 200, 2000, 10,000, or 20,000 nM. After a 3-day incubation, 5 mg/mL of MTT solution (Gibco, Thermo Fisher Scientific, Waltham, MA, USA) was introduced and incubated for 2 h at 37 °C. The supernatant was discarded and dimethyl sulfoxide (Merck Millipore, Darmstadt, Germany) was added. Optical densities at 550 and 650 nm were quantified using a microplate reader (Molecular Devices, San Jose, CA, USA). The cell viability was determined as the percentage of viable cells. The IC_50_ values were calculated using an IC_50_ online calculator (https://www.aatbio.com/tools/ic50-calculator (accessed on 20 August 2025)). Each condition was evaluated in duplicate and three independent MTT assays were conducted.

### 4.7. Growth Curve and Population Doubling Time

Parental PC-9, PC-9/ER, and PC-9/GB cells were trypsinized and seeded at a density of 2 × 10^4^ cells/well in duplicate, in 12-well plates (Corning Inc., Corning, NY, USA). Cells were harvested and counted daily for six consecutive days using trypan blue staining. Growth curves were plotted and the population doubling time was calculated using the following formula:

$$Doubling\;time=\frac{Duration\cdot ln(2)}{\ln\left(\frac{Final\;concentration}{Initial\;concentration}\right)}$$

### 4.8. Colony Formation

The cells were seeded at a density of 2 × 10^3^ cells/well in a 6-well plate and incubated overnight. The media containing different concentrations of 0.05, 0.1, and 0.2 µM for ER and 0.05, 0.5, and 1.0 µM for GB were replaced and cultured for 3 days. Subsequently, the drug-containing media were replaced with drug-free media, and the cells were further incubated for an additional 2 weeks. The medium was removed and colonies were fixed using 100% methanol (Corning Inc., Corning, NY, USA), stained with 0.5% *w*/*v* crystal violet (Sigma-Aldrich, St. Louis, MO, USA), and prepared in 25% methanol (Corning Inc., Corning, NY, USA). The colony formation was quantified.

### 4.9. Cell Analysis by Flow Cytometry

Parental PC-9 and their resistant cells (PC-9/ER and PC-9/GB) were seeded in T25 flasks at a density of 5 × 10^5^ cells and incubated for 24 h. ER was added at concentrations of 0.05, 0.1, and 0.2 μM, whereas GB was added at concentrations of 0.05, 0.5, and 1 μM to both parental and resistant cells, respectively. After a 3-day incubation at 37 °C in 5% CO_2_, the cells were trypsinized, and 1 × 10^6^ cells were pelleted via centrifugation at 700× *g* for 5 min. For apoptosis analysis, the cells were washed with 400 µL of PBS and resuspended in 65 µL of 1× binding buffer. Four microliters of Annexin V-FITC (BD Biosciences, San Jose, CA, USA) and 4 µL of propidium iodide (PI; BD Biosciences, San Jose, CA, USA) were added, and the cells were incubated at room temperature for 30 min in the dark. For cell cycle analysis, after 3 days of incubation, the medium was discarded and the cells were incubated with fresh medium containing 1% FBS for an additional 24 h to induce starvation. A total of 1 × 10^6^ cells were fixed in 1 mL of ice-cold 70% ethanol (JT Baker, Phillipsburg, NJ, USA) and preserved overnight at −20 °C. The fixed cells, following centrifugation, were rinsed with 400 µL of PBS and resuspended in 65 µL of 1× binding buffer. Subsequently, 100 µL of the cell suspension (2 × 10^5^ cells) was transferred to a new tube, combined with 4 µL of PI (BD Biosciences, San Jose, CA, USA) and 1 µL of RNase A (10 mg/mL; Vivantis, Selangor, Malaysia), and incubated at room temperature for 30 min in the dark. Samples were analyzed using the Amnis^®^ ImageStream^®^X Mk II (Luminex Corporation, Austin, TX, USA). Data from Annexin V/PI staining and the percentage of cells in each phase of the cell cycle were analyzed using IDEAS software (version 6.2; Luminex Corporation, Austin, TX, USA).

### 4.10. Western Blotting

Cells were lysed using RIPA buffer (pH 7.4) (Merck Millipore, Darmstadt, Germany) supplemented with a protease inhibitor cocktail (Merck Millipore, Darmstadt, Germany). A dilution of 1:8000 was prepared using distilled water. Total protein yield was measured using the Bradford protein assay (Bio-Rad Laboratories, Hercules, CA, USA). Proteins were separated via 12% sodium dodecyl sulfate-polyacrylamide gel electrophoresis (SDS-PAGE) and subsequently transferred onto a nitrocellulose membrane (Bio-Rad Laboratories, Hercules, CA, USA) at 4 °C overnight. The cell lysate was applied at 50 μg/well, whereas the serum dilution was loaded at 5 µg/well. After blocking with 3% bovine serum albumin (Merck Sigma–Aldrich, St. Louis, MO, USA) for 2 h at room temperature, the membranes were probed with primary antibodies at the following dilution of 1:500 for PARP, cleaved PARP, caspase-9, caspase-3, cleaved caspase-3, caspase-7, and cleaved caspase-7 (Cell Signaling Technology, Danvers, MA, USA), 1:2000 for Cytokeratin 13 and *CEACAM5* (Affinity Biosciences, Cincinnati, OH, USA), and 1:3000 for β-actin (Cell Signaling Technology, Danvers, MA, USA). The membranes were then incubated with horseradish peroxidase-conjugated anti-mouse (Cell Signaling Technology, Danvers, MA, USA) or anti-rabbit (Cell Signaling Technology, Danvers, MA, USA) antibodies at a dilution of 1:2000 at room temperature for 2 h. The bands were detected using Clarity Western ECL substrate (Bio-Rad Laboratories, Hercules, CA, USA). Images were acquired using an ImageQuant LAS 4000 digital imaging system (GE Healthcare, Little Chalfont, UK). The relative expression of each protein in the cells was determined using β-actin as a loading control.

### 4.11. RT-qPCR

Primer sequences for the target genes are listed in Table 4. Primers were designed using Primer3Plus (https://www.bioinformatics.nl/cgi-bin/primer3plus/primer3plus.cgi (accessed on 20 May 2025)). Total RNA was extracted from parental and resistant cells (1 × 10^6^ cells) using RNeasy Plus Mini Kits (Qiagen, Valencia, CA, USA), following the manufacturer’s protocol. The quality and quantity of RNA were assessed using agarose gel electrophoresis and a NanoDrop^®^ ND-1000 UV-Vis spectrophotometer (Thermo Scientific, Waltham, MA, USA). Complementary DNA (cDNA) was synthesized from 1 µg of total RNA using a cDNA synthesis kit (Bio-Rad Laboratories, Hercules, CA, USA) in a Thermal Cycler (Bio-Rad Laboratories, Hercules, CA, USA). Gene expression was calculated using the comparative cycle threshold (2^−ΔΔCt^) method, with GAPDH as the normalization control. Reactions were performed on a Thermal Cycler under the following conditions: initial denaturation at 95 °C for 2 min, followed by 39 cycles of 95 °C for 15 s, and annealing at 60 °C for *KRT13*, *EGR1*, *TLR4*, *SERPINE1*, *FN1*, *CA12*, and GAPDH; 58 °C for *S100A8*; and 57 °C for *CEACAM5* and *FOS*, each for 30 s (Table 4).

### 4.12. Statistical Analysis

All experiments were conducted in duplicate, and data are presented as mean ± standard deviation (SD). An independent t-test was used to compare the two groups. Statistical significance was set at *p* < 0.05. Statistical analyses were conducted using the GraphPad Prism software (version 5.0; GraphPad Software, Inc., La Jolla, CA, USA).

## 5. Conclusions

Integrative analysis of transcriptome data from sensitive PC-9 cells, in comparison with PC-9/ER or PC-9/GB cell lines, identified *CEACAM5* as a common gene, exhibiting different expression patterns; it was downregulated in PC-9/ER and upregulated in PC-9/GB. The expression level of *CEACAM5* may be linked to ER and GB resistance. These findings indicate that *CEACAM5* can be used as a biomarker for predicting the therapeutic response of patients with lung ADC to ER and GB treatments.

## Figures and Tables

**Table 1:** Data information included in the dataset.

BioProject	GEO Dataset	Drug	Platform	IC_50_
PRJNA1050239 [18]	GSE249721	Erlotinib	NextSeq 550	FC = 9.67 PC-9 = 0.06 µM PC-9/ER = 0.58 µM
PRJNA906634 [13]	GSE219044	Erlotinib	NextSeq 2000	FC = 136 PC-9 = 0.012 µM PC-9/ER = 1.68 µM
PRJNA1024457 [19]	GSE244730	Gefitinib	HiSeq 2500	FC = >600 PC-9 = <0.01 µM PC-9/GB = 6 µM
PRJNA530546 [14]	GSE129221	Gefitinib	HiSeq 2000	FC = 256 PC-9 = 0.02 µM PC-9/GB = 5.3 µM
PRJNA304927 [15]	GSE75602	Gefitinib	HiSeq 2500	FC = >50 PC-9 = 0.02 µM PC-9/GB = >1 µM

Abbreviations: IC_50_, concentration inhibiting 50% of cell growth; FC, fold change in resistance.

**Table 2:** Differential expression of 10 hub genes in PC-9 compared to PC-9/ER cell lines.

**Up-Regulated Gene**	**Symbol**	**GSE219044**		**GSE249721**	
		**Log_2_FC**	***p*-adj**	**Log_2_FC**	***p*-adj**
ENSG00000170345	FOS	3.08	4.81 × 10^−16^	4.92	3.88 × 10^−16^
ENSG00000136869	TLR4	4.87	3.04 × 10^−3^	4.23	5.03 × 10^−4^
**Down-regulated gene**	**Symbol**	**GSE219044**		**GSE249721**	
		**Log_2_FC**	***p*-adj**	**Log_2_FC**	***p*-adj**
ENSG00000122786	CALD1	−2.17	4.45 × 10^−64^	−1.69	3.57 × 10^−5^
ENSG00000187498	COL4A1	−2.66	4.02 × 10^−17^	−3.24	4.27 × 10^−3^
ENSG00000149591	TAGLN	−1.85	5.45 × 10^−3^	−2.52	4.55 × 10^−2^
ENSG00000130635	COL5A1	−3.58	2.11 × 10^−137^	−2.36	8.50 × 10^−3^
ENSG00000128591	FLNC	−3.87	1.71 × 10^−169^	−2.40	4.52 × 10^−4^
ENSG00000115414	FN1	−3.82	9.52 × 10^−150^	−5.67	5.14 × 10^−15^
ENSG00000106366	SERPINE1	−6.30	3.65 × 10^−243^	−2.11	4.15 × 10^−3^
ENSG00000137801	THBS1	−3.01	5.63 × 10^−248^	−2.04	3.26 × 10^−2^

Abbreviation: Log_2_FC, log base 2-fold change, *p*-adj, adjusted *p*-values.

**Table 3:** Differential expression of 10 hub genes in PC-9 compared to PC-9/GB cell lines.

**Up-Regulated Gene**	**Symbol**	**GSE75602**		**GSE129221**		**GSE244730**	
		**Log_2_FC**	***p*-adj**	**Log_2_FC**	***p*-adj**	**Log_2_FC**	***p*-adj**
ENSG00000198074	AKR1B10	1.98	1.29 × 10^−9^	2.59	1.44 × 10^−46^	2.25	3.76 × 10^−195^
ENSG00000074410	CA12	3.75	2.05 × 10^−12^	1.70	3.51 × 10^−6^	2.52	1.99 × 10^−26^
ENSG00000104267	CA2	4.81	1.76 × 10^−7^	1.63	2.71 × 10^−12^	3.99	2.26 × 10^−46^
ENSG00000162949	CAPN13	1.64	1.41 × 10^−6^	2.31	8.85 × 10^−12^	3.11	3.91 × 10^−33^
ENSG00000105388	CEACAM5	4.76	5.04 × 10^−75^	6.66	1.94 × 10^−76^	2.07	2.44 × 10^−16^
ENSG00000171401	KRT13	6.11	2.69 × 10^−105^	1.56	2.50 × 10^−5^	3.00	9.33 × 10^−27^
ENSG00000166831	RBPMS2	4.10	2.62 × 10^−15^	1.95	1.09 × 10^−14^	3.55	6.68 × 10^−22^
ENSG00000143546	S100A8	2.46	2.68 × 10^−7^	2.13	4.30 × 10^−19^	4.77	2.36 × 10^−4^
ENSG00000204616	TRIM31	2.39	4.96 × 10^−6^	4.28	2.68 × 10^−79^	4.26	3.33 × 10^−80^
**Down-regulated gene**	**Symbol**	**GSE75602**		**GSE129221**		**GSE244730**	
		**Log_2_FC**	***p*-adj**	**Log_2_FC**	***p*-adj**	**Log_2_FC**	***p*-adj**
ENSG00000120738	EGR1	−3.39	4.65 × 10^−8^	−2.02	3.14 × 10^−25^	−2.25	8.5 × 10^−67^

Abbreviation: Log_2_FC, log base 2-fold change, *p*-adj, adjusted *p*-values.

**Table 4:** RT-PCR primer sequences.

Gene	Primer Sequence	Annealing Temperature (°C)	Product Size (bp)
*KRT13*	F: GACCGCCACCATTGAAAACAA	60	177
	R: TCCAGGTCAGTCTTAGACAGAG		
*CEACAM5*	F: CTGTCCAATGACAACAGGACC	57	174
	R: ACGGTAATAGGTGTATGAGGGG		
*EGR1*	F: ACCCCTCTGTCTACTATTAAGGC	60	83
	R: TGGGACTGGTAGCTGGTATTG		
*TLR4*	F: AGACCTGTCCCTGAACCCTAT	60	147
	R: CGATGGACTTCTAAACCAGCCA		
*FOS*	F: GGGGCAAGGTGGAACAGTTAT	57	126
	R: CCGCTTGGAGTGTATCAGTCA		
*SERPINE1*	F: AGTGGACTTTTCAGAGGTGGA	60	151
	R: GCCGTTGAAGTAGAGGGCATT		
*FN1*	F: AGGAAGCCGAGGTTTTAACTG	60	106
	R: AGGACGCTCATAAGTGTCACC		
*CA12*	F: AGTGACATCCTCCAGTATGACG	60	164
	R: GTGGCACTGTAGCGAGACT		
*S100A8*	F: ATTTCCATGCCGTCTACAGG	58	170
	R: TGCCACGCCCATCTTTATCA		
*GAPDH*	F: CCACAGTCCATGCCATCAC	60	451
	R: TCCACCACCCTGTTGCTGTA		

## Data Availability

The original contributions presented in this study are included in the article. Further inquiries can be directed to the corresponding author.

## Abbreviations

The following abbreviations are used in this manuscript:

ADC	Adenocarcinoma
ATCC	American Type Culture Collection
*CEACAM5*	Carcinoembryonic antigen-related cell adhesion molecule 5
cDNA	Complementary DNA
DAVID	Database for Annotation Visualization and Integrated Discovery
DEGs	Differentially expressed genes
DMSO	Dimethyl sulfoxide
EGFR	Epidermal growth factor receptor
EGFR-TKIs	EGFR Tyrosine Kinase Inhibitors
ER	Erlotinib
FBS	Fetal bovine serum
GB	Gefitinib
GEO	Gene Expression Omnibus
GO	Gene Ontology
IC_50_	Half maximal inhibitory concentration
KEGG	Kyoto Encyclopedia of Genes and Genomes
*KRT13*	Cytokeratin 13
MTT	3-(4,5-dimethylthiazol-2-yl)-2,5-diphenyltetrazolium bromide
NSCLC	Non-small cell lung cancer
PARP	Poly (ADP-ribose) polymerase
PBS	Phosphate-buffered saline
PC-9/ER	ER-resistant cell line
PC-9/GB	GB-resistant cell line
qRT-PCR	Quantitative reverse transcription polymerase chain reaction
RNA-seq	RNA sequencing
RPMI	Roswell Park Memorial Institute
SD	Standard deviation
SDS	Sodium dodecyl sulfate–polyacrylamide gel electrophoresis (SDS-PAGE)
